# Supplementary material for: Patterns of Intron Gain and Loss in Fungi
Source: PLoS Biol. 2004 Nov 30;2(12):e422. doi: 10.1371/journal.pbio.0020422 (PMC532390; doi:10.1371/journal.pbio.0020422)
Supplement: Table S1 — Also available at http://genes.mit.edu/NielsenEtAl/. (4.3 MB ZIP). [file pbio.0020422.st001.zip › NielsenEtAl/html/935.html]

AN7185.1.NCU09202.1.MG10596.1.FG02795.1


```
 CLUSTAL W (1.82) Multiple Sequence Alignments - Introns Inserted


Sequence 1: MG10596.1	582 aa
Sequence 2: FG02795.1	510 aa
Sequence 3: NCU09202.1	578 aa
Sequence 4: AN7185.1	581 aa
Alignment Length: 615 aa
Number Identitical Residues: 357 aa
Alignment Score (without introns) 15543


MG10596.1 	MLPARAQSSNAFPPTEPKPSQAYASSQPSREPAHSALGQGLDWTGQDTPPSQRNPHRPPP
NCU09202.1	----------MNGSQQAKDSTAQTSAQVASSPAATASNGNLNKNSSSNTNNKRKKDGLKP
FG02795.1 	------------------------------------------------------------
AN7185.1  	------------MEGVDFQSVLNKGKQMASNVAATATNG-------STNKKRRKGTDLKP
          	                   :    . . : . : :: .        ..  .  .     .

MG10596.1 	RCFEIHAPR~RSK2TAHLSAH~SPTSSS----SHDDPENTADEEDSEDYCKGGYHPVQIG
NCU09202.1	-IITTDGPG2AAH~LASAMTN~SPTSSSSA--AEDIAENTADEEDQEDYCKGGYHPVTIG
FG02795.1 	---------~---~----MAH~SPSSSS----VEDAAENTADEEDSEDYCKGGYHPVQVG
AN7185.1  	IVTSDSATP~ADQ~TGPTSDN2VPPSRSGSSSSEEEIETTAEEEDSEDYCKGGYHPVQIG
          	      ..       .    :  *.* *.::: .:  *.**:***.*********** :*

MG10596.1 	EKFKDGKYTIVRKLGWGHFSTVWLSRDNSSGKHVALKVVRSAAHYTETAIDEIKLLNKIV
NCU09202.1	EKFKDGKYTVVRKLGWGHFSTVWLSRDNTTGKHVALKVVRSAAHYTETAIDEIKLLNKIV
FG02795.1 	EKFKDGKYTVVRKLGWGHFSTVWLSRDNTNGKHVALKVVRSAAHYTETAIDEIKLLNKIV
AN7185.1  	ELYNNGRYVVVRKLGWGHFSTVWLSRDTTTGKHVALKVVRSAAHYTETAIDEIKLLNRIV
          	* :::*:*.:*****************.:.***************************:**

MG10596.1 	QAKPDHPGRKHVVSLLDSFDHKGPNGTHVCMVFEVLGENLLGLIKKWNHRGIPMALVKQI
NCU09202.1	QANPNHPGRRHVVSLLDSFEHKGPNGTHVCMVFEVLGENLLGLIKRWNHRGIPMALVKQI
FG02795.1 	QAKPDHPGRKHVVSLLDSFEHKGPHGTHVCMVFEVLGENLLGLIKRWNHRGIPMPLVKQI
AN7185.1  	QANPSHPGRKHVVSLLDSFEHKGPHGVHVCMVFEVLGENLLGLIKKWNHRGIPMPLVKQI
          	**:*.****:*********:****:*.******************:********.*****

MG10596.1 	TKQVLLGLDYLHRECGIIHTDLKPENVLIEIGDVEQIVKRVVKSEPS--DKENNRNGRRR
NCU09202.1	TKQVLLGLDYLHRECGIIHTDLKPENVLIEIGDVEQTVKRVVKDEPN--DKENTRNSRRR
FG02795.1 	TKQVLLGLDYLHRECGIIHTDLKPENVLIEIGDVEQIVKRVVKPEAA--EKENNRNGRRR
AN7185.1  	TKQVLLGLDYLHRECGIIHTDLKPENVLIEIGDVEQIVKTYVKEEAKKEQKEDNRNGRRR
          	************************************ **  ** *. ..:**:.**.***

MG10596.1 	RRTLITGSQPLPSPLNATFNHANLFPSPSSQHSSLGQMLHDG1AK~SKSSTSPRR-----
NCU09202.1	RRTLITGSQPLPSPLNASFNQGSMFPSPAP--QSLGQMLAEG1AK~SQEGSPFHNK----
FG02795.1 	RRTLITGSQPLPSPLNTSFNQSNLFPSPTP-HSSLAGVLNDG1-K~SSNEASPK------
AN7185.1  	RRTLITGSQPLPSPLNTTFEFKHSSQNSHS---SLSQVINES~PG1TSEAPSMRQLLGIK
          	****************::*:      .. .   **. :: :. .  :.. .. :   . .

MG10596.1 	DGDDKQKQREKSA2---------------DLLSREVSGISLDKT-------GEKRKADDN
NCU09202.1	NGEDDQSRREKSA~---------------DLLSKEVSGISLDKTATPPATSGDKRNLDDM
FG02795.1 	PSDDAQKQREKSA2---------------DLLSREVSGISLDKSNSPSTAAGDKRKAEDA
AN7185.1  	DEDEQQKQREKTA~YVERLLEGTSLTDDSDLLEREVSGISLDKP-----------SSDED
          	  :: *.:***:*   .   ..:: :..:***.:*********.           . :: 

MG10596.1 	HGFDVISVKIADLGNACWVSHHFTNDIQTRQYRSPEVILGSKWGASTDVWSMAAM0VFEL
NCU09202.1	Q-FDIISVKIADLGNACWVNHHFTNDIQTRQYRSPEVILGAKWGASTDVWSMAAM0VFEL
FG02795.1 	HAFDVISVKIADLGNACWVNHHFTNDIQTRQYRSPEVILGSKWGASTDVWSMAAM0VFEL
AN7185.1  	IDCGIISVKIADLGNACWVGHHFTNDIQTRQYRSPEVILGSKWGASTDIWSMACM0VFEL
          	   .:**************.********************:*******:****.* ****

MG10596.1 	ITGDYLFDPQSGTKYGKDDDHIAQIIELLGPFPKSLCLSGKWSQEIFNRKGELRNIHRLR
NCU09202.1	ITGDYLFDPQSGTKYGKDDDHIAQIIELLGAFPKSLCLSGKWSQEIFNRKGELRNIHRLR
FG02795.1 	ITGDYLFDPQSGTKYGKDDDHIAQIIELLGPFPKSLCLSGKWSQEIFNRKGELRNIHRLR
AN7185.1  	ITGDYLFDPQSGTRYGKDDDHIAQVIELLGPFPKSLCLSGRWSQEIFNRKGELRNIHRLR
          	*************:**********:*****.*********:*******************

MG10596.1 	HWALPDVLKEKYHFKEEEAKRIAEFLLPMLELVPEKRANAGGMAAHLWLDDTPGMKGVKI
NCU09202.1	HWALPDVLREKYHFKAEEAQRIADFLMPMLELIPERRANAGGMAGHSWLEDTPGMKGIKI
FG02795.1 	HWALTDVLREKYHFKEEEAKRIADFLTPMLELVPDKRANAGGMAGHNWLEDTPGMKGLKI
AN7185.1  	HWSLPDVLREKYHFSVAQAKAISDFLLPMLEVLPERRANAGGMASHEWMKDTRGMQEVDL
          	**:*.***:*****.  :*: *::** ****::*::********.* *:.** **: :.:

MG10596.1 	PGLDAGSRGEGIDGWANEVRKR
NCU09202.1	DKVEVGSRGEGIEGWASEVRKR
FG02795.1 	EGLEVGGRGEGIDGWSTEVRKR
AN7185.1  	G-LTPGSRGEGIEGWATEVKRR
          	  :  *.*****:**:.**::*
```
